# The Antiviral Role of Galectins toward Influenza A Virus Infection—An Alternative Strategy for Influenza Therapy

**DOI:** 10.3390/ph14050490

**Published:** 2021-05-20

**Authors:** Chih-Yen Lin, Zih-Syuan Yang, Wen-Hung Wang, Aspiro Nayim Urbina, Yu-Ting Lin, Jason C. Huang, Fu-Tong Liu, Sheng-Fan Wang

**Affiliations:** 1 Center for Tropical Medicine and Infectious Disease, Kaohsiung Medical University, Kaohsiung 80708, Taiwan; pigpipi831205@gmail.com (C.-Y.L.); r99100125@gmail.com (Z.-S.Y.); bole0918@gmail.com (W.-H.W.); aspiro.urbina@hotmail.com (A.N.U.); 2 Department of Medical Laboratory Science and Biotechnology, Kaohsiung Medical University, Kaohsiung 80708, Taiwan; kin2092514@gmail.com; 3 Division of Infectious Disease, Department of Internal Medicine, Kaohsiung Medical, University Hospital, Kaohsiung Medical University, Kaohsiung 80708, Taiwan; 4 Department of Biotechnology and Laboratory Science in Medicine, National Yang-Ming University, Taipei 112304, Taiwan; jchuang2@ym.edu.tw; 5 Institute of Biomedical Sciences, Academia Sinica, Taipei 11529, Taiwan; ftliu@ibms.sinica.edu.tw; 6 Department of Laboratory Medicine, Kaohsiung Medical University Hospital, Kaohsiung Medical University, Kaohsiung 80708, Taiwan

**Keywords:** galectins, influenza A virus, carbohydrate recognition domain, anti-influenza, review, PRISMA

## Abstract

Animal lectins are proteins with carbohydrate recognition activity. Galectins, the β-galactoside binding lectins, are expressed in various cells and have been reported to regulate several immunological and physiological responses. Recently, some galectins have been reported to regulate some viral infections, including influenza A virus (IAV); however, the mechanism is still not fully understood. Thus, we aim to review systemically the roles of galectins in their antiviral functions against IAVs. The PRISMA guidelines were used to select the eligible articles. Results indicated that only Galectin-1, Galectin-3, and Galectin-9 were reported to play a regulatory role in IAV infection. These regulatory effects occur extracellularly, through their carbohydrate recognition domain (CRD) interacting with glycans expressed on the virus surface, as well as endogenously, in a cell–cell interaction manner. The inhibition effects induced by galectins on IAV infection were through blocking virus–host receptors interaction, activation of NLRP-3 inflammasome, augment expression of antiviral genes and related cytokines, as well as stimulation of Tim-3 related signaling to enhance virus-specific T cells and humoral immune response. Combined, this study concludes that currently, only three galectins have reported antiviral capabilities against IAV infection, thereby having the potential to be applied as an alternative anti-influenza therapeutic strategy.

## 1. Introduction

Influenza is an acute, contagious infectious disease caused by the influenza virus. Influenza viruses belong to the Orthomyxoviridae family, with six to eight segments of linear negative-sense, single-stranded RNA and comprise a family of four distinct viruses (influenza A, B, C, and D viruses) [1]. Among them, influenza A and B viruses are the pathogens causing seasonal influenza disease. The seasonal influenza virus epidemics are estimated to cause 3–5 million cases of severe infection and result in 290,000–650,000 deaths annually worldwide [2,3]. Higher mortality rates are seen in the elderly over 65 years old and children under 5 years old, as well as people in developing countries [4]. The burden for seasonal influenza epidemics is substantial and is significantly increased during influenza pandemics, such as a more recent, swine-originated H1N1 influenza pandemic in 2009 [5].

Great improvement has been achieved in the treatment of influenza, especially for patients with severe influenza infection. To date, the most effective strategies for the prevention and control of influenza disease are vaccination and antiviral therapy. Although influenza vaccine and antiviral drugs greatly reduced influenza outbreaks, there are still many limitations to these two approaches [6,7,8]. The current influenza vaccine can be either trivalent or quadrivalent, which may contain the strains of seasonal circulating influenza A (H1N1, H3N2) and influenza B (Yamagata or Victoria lineage). A substantial challenge for influenza vaccine is the continuous viral antigenic changes and variations, which may lead to vaccine mismatch that results in the reduction in the vaccine’s effectiveness (protection rate from 10 to 60%) [9,10]. As to influenza antiviral drugs, the major candidates are the inhibitors that can block NA activity and M2 protein function to ameliorate influenza release and viral uncoating during the influenza virus life cycle. The most common clinical treatment of influenza are NA inhibitors including, oseltamivir, zanamivir, and peramivir, as well as the M2 proton channel blockers including amantadine and rimantadine. Unfortunately, several emerging strains of influenza A virus, such as the 2009 H1N1 influenza virus and some avian H5N1 or H7N9 isolates, were reported to be naturally carrying drug-mutation genes, which leads to resistance against these drugs [11]. 

Lectins are a group of proteins with carbohydrate recognition activity, and they are characterized into several families based on their different cellular locations and their specificities for a variety of carbohydrate structures according to the features of their carbohydrate recognition domain (CRD) binding preference. Interaction between lectins and their recognition glycans mainly relies on their CRD binding to certain particular oligosaccharides expressed on cellular or microbial surfaces. Lectins have been categorized into many families based on their conserved structure of sequence motifs for sugar-binding and carbohydrate specificities such as calnexin, DC-SIGN, L-SIGN, mannose-6-phosphate receptors (MPRs), siglecs, galectins, and intelectins [12,13]. Recently, several lectins have been reported to participate in the regulation of virus infection and replication [14,15]. 

**Figure 1:**
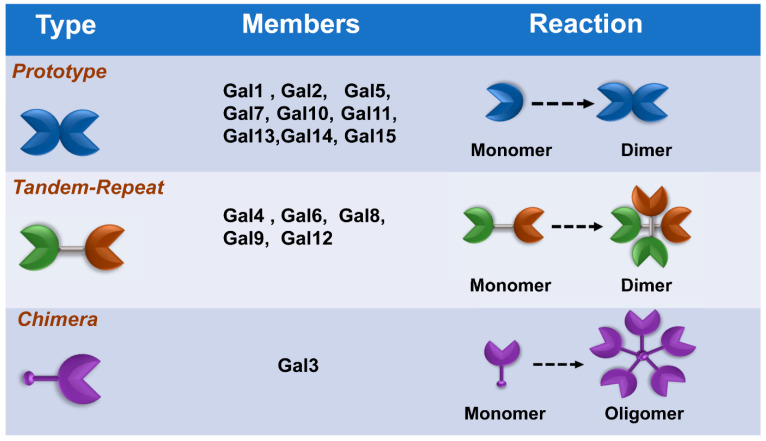
Classification of galectins. According to the number and arrangement of the carbohydrate recognition domains (CRDs), galectin family members are classified into three main types: prototype, chimera type, and tandem-repeat type. Some galectins can self-associate into dimers or oligomers.

Galectins, previously categorized as S-type lectin, have a preference to bind β-galactoside sugars, such as N-acetyllactosamine (Galβ1-3GlcNAc or Galβ1-4GlcNAc), which are presented in N-linked and O-linked glycoproteins [16,17]. Galectins are the most conserved and ubiquitous lectin family, detected from protists to mammals. The first galectin was identified and characterized from the electric organs of the electric eel, *Electrophorus electricus* [18], and since then, members of the galectin family have been found in mammals, birds, amphibians, fish, nematodes, sponges, and some fungi [19,20]. Galectins have been reported to regulate various cellular or immunological functions [21]. Galectins are reported to be the pattern recognition receptors (PRRs) to recognize microbial invasion and regulate innate immune responses [22]. The basic structure of a galectin contains a carbohydrate recognition domain (CRD) (about 130 amino acids) that connects to a tandem repeat domain. Currently, galectins have been categorized into three main types—prototype, chimera type, and tandem-repeat type. The prototype galectins (Gal-1, Gal-2, Gal-5, Gal-7, Gal-10 Gal-11, Gal-13, Gal-14, and Gal-15) contain one CRD and are either monomers or noncovalent homodimers; the unique chimera-type galectin (Gal-3) contains a rare tandem that repeats of proline and glycine-rich short stretches fused onto the CRD; and tandem-repeat galectins (Gal-4, Gal-6, Gal-8, Gal-9, and Gal-12) contain two distinct CRDs, in tandem, connected by a linker peptide. Currently, 15 galectins have been identified in mammals, whereas 12 galectins are detected in humans [21,23] (Figure 1). 

Recently, galectins have been reported to participate in the regulation of several viral infections and replications, such as HIV-1, enterovirus, HSV-1, adenovirus, and dengue viruses. Some reports indicated that galectins have abilities to ameliorate influenza virus infection; however, the details of the role and regulatory mechanism are still not fully understood [17,24,25]. Accordingly, this study performs a systemic literature review to analyze and summarize the potential anti-influenza function of galectins.

## 2. Methods

**Figure 2:**
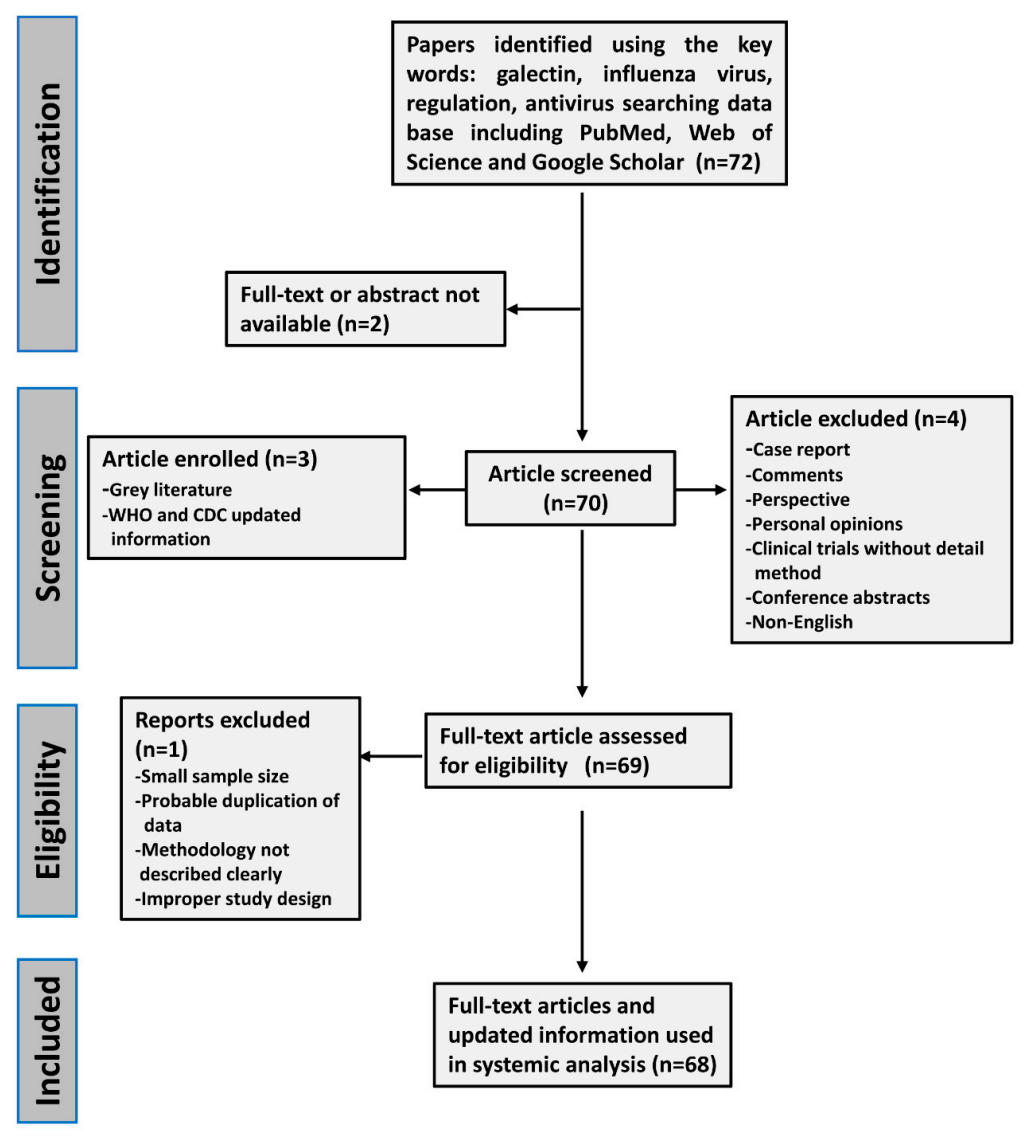
The flow chart of the criteria and selection standard for included literature in this systemic review. The eligible references were selected according to PRISMA guidelines. Two independent reviewers assessed the level of data quality from the selected studies. Disagreements were resolved by joint discussion and consensus.

In this study, we analyzed the information from references regarding the antiviral function of galectins against influenza virus infections. The systemic review was performed following the PRISMA guidelines to select eligible articles [26]. References were selected from several databases including PubMed, Web of Science, and Google Scholar by using search strings containing a combination of terms that included influenza virus, antiviral, therapy, and galectins. Search results were limited to articles published in the English language. The selected articles were reviewed to assess their relevance, case numbers, and quality of methodology. Two independent reviewers assessed the level of data quality from the selected studies. Disagreements were resolved by joint discussion and consensus. Ethics approval and informed consent were not required for this study (Figure 2).

## 3. Results

### 3.1. Galectin-1 (Gal-1)

#### 3.1.1. Basic Information of Gal-1

Galectin-1(Gal-1) was the first protein identified in this family. Gal-1 acts typically as a pro-resolving mediator by repressing a number of innate and adaptive immune programs. Gal-1 is encoded by the *LGALS1* gene, which is located on chromosome 22q12. Gal-1 is categorized into the prototype of galectins and is composed of two subunits of 14.5 kDa (135 aa) present in a dynamic dimerization equilibrium. Gal-1 is structurally formed as a monomer or homodimer with a noncovalent linkage. These structural formations are linked with distinctive biological activities. Gal-1 recognizes multiple galactose-β1-4–N-acetyl-glucosamine (N-acetyl-lactosamine [LacNAc]) units present on the branches of N- or O-linked glycans on diverse cell surface receptors. Gal-1 could be detected both in the outside and inside of cells and is expressed in several different types of cells and tissues [27]. 

#### 3.1.2. Gal-1 Blocks the Interaction between Influenza Virus and Sialic Acid Receptors

Blocking virus interactions with its corresponding receptor is a key step to inhibit virus infection. Regarding the influenza virus, the glycoproteins, HA and NA, are major determinants in the pathogenicity of influenza infection. Furthermore, HA is the main protein that interacts with α-2,3 or α-2,6 sialic acid-containing glycans on the host cell surface, subsequently inducing viral internalization and penetration of the viral genome through membrane fusion. With this aim in mind, Yang et al. indicated that Gal-1 could bind to HA protein of influenza A/WSN/33 virus and subsequently inhibited virus infection from in vitro assays. They proposed a potential mechanism in which Gal-1 directly binds to the HA envelope glycoproteins and restricts the viral infectivity as well as hemagglutination activity [28]. However, the inhibitory capabilities of Gal-1 on IAVs could be abrogated in IAV-infected Gal-1 knockout (KO) mice, compared to the wild-type mice. With the exception of H1N1, this study also indicated that Gal-1 could interact with other subtypes of IAVs, such as H2N2, H3N2, H5N2, and H6N1 [28]. A similar observation was reported by Bao et al. since their study indicated that infection with the 2009 swine-original influenza A (H1N1) subtype (H1N1pdm09) induced Gal-1 expression in both A549 cells and mouse bronchoalveolar lavage fluid (BALF), and expression of Gal-1 ameliorated H1N1pdm09-induced acute lung injury [25]. Combined, these studies suggested that Gal-1 expression or treatment inhibited IAV infection and virus-induced lung injury. However, although most studies reported the inhibitory effect of Gal-1 on IAVs, a previous study indicated that Gal-1 promoted influenza A/NIB/23/89 MA (H1N1) virus, binding to MDCK cells in a dose-dependent manner, but Gal-1 did not have any effect on virus internalization [29]. 

#### 3.1.3. Gal-1 Participates in Influenza Virus-Induced Cell Apoptosis

Understanding the pathogenic molecular mechanisms and pathogenicity is essential for the control, prevention, and treatment of influenza diseases. Previous research studying the pathogenesis and disease mechanisms of cell damage induced by the invasion of the H1N1pdm09 virus indicated that Gal-1 could be upregulated in H1N1pdm09 virus-infected human bronchial epithelial cells (BEAS-2B). This study also indicated that Gal-1 participated in the regulation of cytopathic processes caused by the H1N1pdm09 virus via interaction with cyclin-dependent kinases (CDKs) and cyclins to induce an arrest of the cell cycle at the G0/G1 phase. This study suggested that Gal-1 may play an important role in H1N1pdm09 virus-induced cell apoptosis [30]. 

#### 3.1.4. Gal-1 Treatment Enhances Immune Response and Ameliorates Influenza Virus-Induced Patho Genesis

Previously, Bao et al. also reported that recombinant Gal-1 (rGal-1) treatment reduced mice fatality via mediating the expression of cytokines and chemokines, including IL-6, IL-10, IL-12(p40), IL-12(p70), IL-1β, MCP-1, G-CSF, MIP-1α, and RANTES in serum and BALF from infected mice, compared with mock treatment groups [25]. The other study performing pathological analysis indicated that rGal-1 treatment significantly reduced viral replication, inflammatory cell infiltration in mouse lungs, and attenuation of apoptosis occurrence from H1N1pdm09-infected mice. Their data suggested that Gal-1 may persist inside the cell to control intracellular processes, or that Gal-1 could be released to the extracellular space to control H1N1pdm09 infection. Yang et al. also reported a similar inhibitory effect by Gal-1 from A/WSN/1933(H1N1) infected mice models [28]. Their results indicated that A/WSN/33(H1N1) infection upregulated Gal-1 in mice, and Gal-1 expression or treatment significantly reduced body weight loss and death rate [28]. In addition, treatment of rGal-1 intranasally significantly ameliorated influenza virus replication, infiltration, and inflammation in infected mice lung, suggesting that Gal-1 treatment reduced influenza induced pathogenicity [28]. 

#### 3.1.5. The Polymorphism of LGALS1 Gene Correlates with Resistance of IAV Infection in Humans

Genetic variation among different races or persons is known to correlate with the susceptibility to infectious disease or disease severity. In addition, influenza susceptibility and resistance in genetic and proteomic aspects in the population pose an important concern. A previous report used a genome-wide association study (GWAS) between avian influenza H7N9-infected patients and heavily exposed healthy poultry workers to identify the important genes that correlated with the susceptibility to H7N9 influenza infection [31]. This study indicated that one of the top SNPs was rs13057866 of *LGALS1* (an SNP 2 kb upstream of *LGALS1*) from a single-SNP analysis. Gal-1 is an essential candidate that contributes to the differential susceptibility to H7N9 influenza. In addition, two biological pathways, including extracellular matrix (ECM)–receptor interaction and mitogen-activated protein kinase (MAPK) signaling are significantly correlated with the susceptibility in humans [31]. 

### 3.2. Galectin-3 (Gal-3)

#### 3.2.1. Basic Information of Gal-3

Galectin-3 (Gal-3) is the only chimera-type galectin. Human galectin-3 is a 31~35 kDa protein that is coded by a single gene, *LGALS3*, located on chromosome 14 [32]. Gal-3 composes of tandem repeats of short proline-rich amino acid segments (approximately 120 amino acids) connected to a C-terminal CRD [33]. The N-terminal domain of galectin-3 is essential for its multimerization, and it is sensitive to proteolysis-by-matrix metalloproteinases and may participate in the interaction with other intracellular proteins. The C-terminal CRD of Gal-3 is responsible for its interaction with glycoconjugates containing N-acetyl-lactosamine. The first 12 amino acids of Gal-3 are essential for its secretion and nuclear translocation [34]. Gal-3 has been reported to have multiple functions, which partially correlate with its localization of the cell. Gal-3 has been reported to participate in various biological activities such as cell growth, pre-mRNA splicing, differentiation, angiogenesis, inflammation, fibrosis, apoptosis, and host defense [35,36,37]. Recently, Gal-3 was reported to regulate certain viral infections, such as herpes simplex virus (HSV), enterovirus, and HIV-1 [16,17]. However, limited information is available regarding Gal-3 in IAV infection.

#### 3.2.2. Gal-3 Expression Augment Expression of Antiviral Genes to Fight against IAV Infection

**Figure 3:**
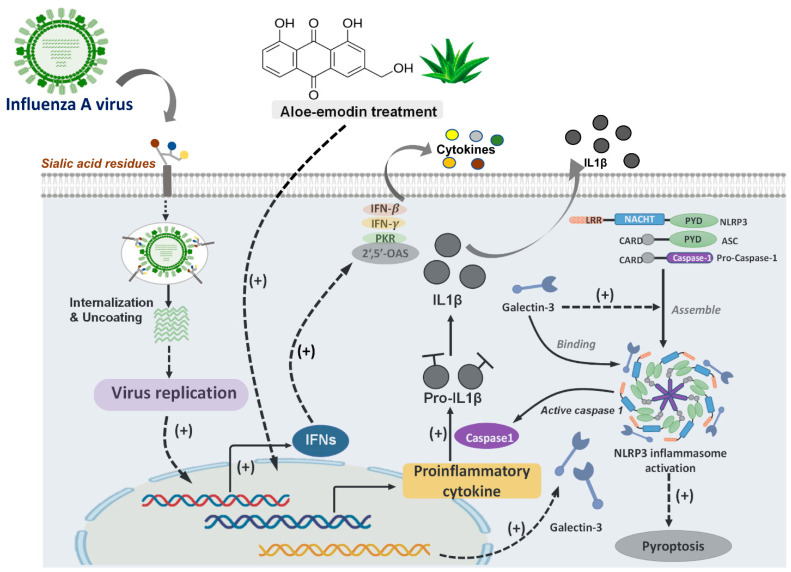
The antiviral role of galectin-3 in influenza A virus infection. The Influenza A virus (IAV) infection resulted in induction of endogenous galectin-3(Gal-3) expression. Gal-3 could bind to NLRP3 inflammasome, and Gal-3/NLRP3 interaction facilitated NLRP3 apoptosis-associated speck-like protein containing a CARD (ASC) inflammasome assembly and ASC oligomerization. These interactions might activate NLRP3 inflammasome, further inducing pyroptosis. In addition, Aloe–emodin treatment could induce Gal-3 expression and activated antiviral gene expression, as well as cytokine production.

Induction of antiviral gene expression and cytokine production against viral replication are immunological strategies to combat IAV infection. Anthraquinone derivatives such as aloe–emodin, emodin, and chrysophanol were previously reported to display antiviral activity against viruses. Accordingly, Li et al. studied the antiviral abilities of aloe–emodin and other anthraquinones on influenza H1N1 virus by in vitro assays. Their data from proteomic analysis and Western blot analysis demonstrated that aloe–emodin treatment upregulated Gal-3 in MDCK cells. Recombinant Gal-3 treatment showed antiviral activity that ameliorated IAVs replication. These results proposed that Gal-3 upregulation is involved in the antiviral mechanism of aloe–emodin against influenza. Furthermore, this study denoted that Gal-3 expression could augment expression of antiviral genes including IFN-β, IFN-γ, PKR, and 2′,5′-OAS via regulating JAK/STAT pathways in the infected cells and proposed that Gal-3 exerted cytokine-like regulatory actions, demonstrating that Gal-3 expression has anti-influenza capabilities [38] (Figure 3).

#### 3.2.3. Gal-3 Participates in the Activation of NLRP3 Inflammasome during IAV Infection

In recent decades, several subtypes of avian influenza virus have been reported to cause human infection, such as H5N1, H5N6, H7N4, H7N9, and H9N2. WHO previously announced that H5N1 and H7N9 avian influenza viruses are the two major avian influenzas that require additional attention and continuous monitoring owing to the severe lung damage and immune dysregulation leading to high mortality in human infection [39]. The nucleotide and oligomerization domain, leucine-rich repeat-containing protein (NLR) family members have been reported to be involved in the intracellular surveillance toward infections and recognition of self-derived damage-associated molecular patterns (DAMPs). Predominantly, the NLRP3 inflammasome has been reported to enhance antiviral immune responses against virus infection [40]. More recently, Chen et al. reported that endogenous Gal-3 regulated H5N1 avian influenza virus-induced lung pathology [41]. This study demonstrated that Gal-3 knockout (KO) mice exhibited less inflammation in the lungs and reduced IL-1β levels in bronchoalveolar lavage fluid, compared to the Gal-3 wild-type (WT) mice after receiving H5N1 influenza inoculation. Further, their results also indicated that endogenous Gal-3 enhanced the H5N1-induced pathogenesis via the promotion of host inflammatory responses and upregulation of IL-1β production from macrophages. Their data demonstrated that Gal-3 interacted with NLRP3 inflammasome and this interaction facilitated NLRP3 apoptosis-associated speck-like protein containing a CARD (ASC) inflammasome assembly and ASC oligomerization, suggesting that Gal-3 plays a role in H5N1-induced inflammation and lung damage [41] (Figure 3).

#### 3.2.4. Gal-3 Ameliorates the Immunopathogenesis of IAVs and *Streptococcus pneumoniae* Coinfection In Vivo

IAVs and *Streptococcus pneumoniae* (*S. pneumoniae*) are two major causes of respiratory tract infections, particularly during coinfection. The coinfection caused a synergistic interaction between these two pathogens and induced a complex network of dysregulated immune responses. Recently, Gal-3 was reported to play a role in IAVs and *S. pneumoniae* coinfection [17]. Nita-Lazar et al. observed that IAVs and *S. pneumoniae* coinfection in mice models demonstrated an upregulation of Gal-1 and Gal-3 expression; further, the binding of secreted Gal-1 and Gal-3 to the epithelial cell surface could modulate the expression of SOCS1 and RIG1, and activate ERK, AKT or JAK/STAT1 signaling pathways. These signaling pathways regulated and enhanced the expression of proinflammatory cytokines including IFN-γ, TNF-α, IL-1β, IL-6, IL-8, IL-10, IL-12, and IL-15, suggesting that the induced Gal-1 or Gal-3 have abilities that lead to protentional hypercytokinemia [42]. This study also indicated that downregulation of SOCS1 expression by Gal-3 would enable a strong cytokine response directed to induce the effective antimicrobial response against *S. pneumoniae* [42]. 

#### 3.2.5. Gal-3 Could Bind to HA of IAVs and Desialylated Airway Epithelial Cells

Airway epithelial cells are the primary targets of the influenza virus, and the infection is mostly confined within the lungs. A previous report studying protein–carbohydrate interactions indicated that sialylated and desialylated multivalent glycoligands could selectively bind, with high affinity, to influenza HA and human Gal-3, respectively, and suggested that the synthetic multivalent glycoligands could efficiently inhibit the attachment of Gal-3 to influenza-susceptible cell lines [43]. Similarly, Lazar et al. indicated IAV-infected airway increased susceptibility to subsequent infection with *Streptococcus pneumoniae*. This may be due to Gal-3 binding to the glycans on influenza HA, facilitating viral binding and further desialylated the airway epithelial cells by neuraminidases from IAVs, subsequently increasing *S. pneumoniae* adhesion [44]. These studies suggested that blocking Gal-3 expression could reduce airway epithelium cell infection prompted by IAVs and reduce the occurrence of subsequent infection with *Streptococcus pneumoniae* [44]. 

### 3.3. Galectin-9 (Gal-9)

#### 3.3.1. Basic Information of Gal-9

Galectin-9 (Gal-9) is a tandem-repeat type galectin. The Gal-9 is encoded by the *LGALS9* (HGNC:6570) gene, which is located on chromosome 17q11.2 and consists of 11 exons encoding a 355 amino acid long protein of approximately 36~39.5 kDa. Gal-9 was initially detected from mouse embryonic kidney and was cloned in 1997 [45]. In humans, two additional *LGALS9*-like genes have been described, *LGALS9B* (HGNC:24842) and *LGALS9C* (HGNC:33874), which are both located on chromosome 17p11.2, close to *LGALS9* [46]. Both have an intact exon–intron structure, but whether these genes are transcriptionally active still remains under debate. Gal-9 has been reported as a T-cell-derived eosinophil-specific chemoattractant to regulate various cellular and immune responses [47,48]. Currently, Gal-9 has been reported to have multiple effects on various viruses including herpes simplex virus, hepatitis B and C virus, respiratory syncytial virus, and HIV-1 [17,49,50]. Similarly, a few studies were available regarding the role of Gal-9 in IAV infection.

#### 3.3.2. Gal-9 Binds to IAVs to Block Virus Attachment

Hattori et al. reported that Gal-9 exhibited anti-influenza function via directly binding to IAVs including A/Puerto Rico/8/34 (H1N1), A/Aichi/2/68 (H3N2), and A/Hong Kong/483/97 (H5N1), further blocking the virus from interacting with the sialic acids receptors on the host cells [51]. In addition, their data also indicated that Gal-9 transgenic mice had a significantly higher survival rate, compared to wild-type mice after being infected with A/Puerto Rico/8/34. This study also noted that IAV infection induced endogenous Gal-9 expression in mice after postinoculation [51].

#### 3.3.3. Gal-9/Tim-3 Signaling Control IAV-Infected Cells

The T-cell-mediated immune response is known to play a key role in the protection and viral clearance during virus infection. T-cell immunoglobulin and mucin-domain (Tim) molecules are the essential regulators in the immune system and are associated with inflammatory conditions. Among them, Tim-3 signaling in Th-1 cells is well-characterized. Gal-9 was previously identified as the ligand of Tim-3 [52]. Gal-9 could bind to Tim-3 in a glycosylation-dependent manner [53]. Currently, the Tim-3 mediated downstream signaling is not fully understood. Gal-9–Tim-3 interaction could activate phosphorylation of the tyrosine motif Y265 (Y256 in mouse) and Y272 (Y263 in mouse) in its cytosolic domain of Tim-3 [54]. Interestingly, Sharma et al. reported that during HK/×31 (H3N2) and A/Puerto Rico/8/34 (H1N1) virus infection, the Tim-3 was upregulated by virus-specific CD8 T cells after IAV Infection, and Tim-3 expressing cells would undergo apoptosis upon exposure to recombinant Gal-9 from in vitro and ex vivo experiments. These results may suggest that Gal-9 has anti-influenza abilities through interacting with Tim-3 [54]. However, their in vivo data demonstrated that Gal-9 knockout (G9KO) mice mounted a more robust acute phase virus-specific CD8 T-cell response, as well as higher and more rapid virus-specific serum IgM, IgG, and IgA responses, further clearing the virus more rapidly than the WT mice after IAV infection, suggesting that endogenous Gal-9 may play the other role to limit the magnitude of CD8 T-cell responses [55]. Combined, these data suggested that extracellular and endogenous Gal-9 may play different roles in the regulation of IAV infection, and manipulation of Gal-9/Tim-3 signaling may represent a convenient approach to improve influenza vaccine. 

#### 3.3.4. Plasma Gal-9 Could Be a Biomarker for IAV Infection

Currently, there is still a lack of specific biomarkers for the monitoring of influenza infection and disease progression. Previously, a clinical cohort study indicated that patients infected with seasonal IAVs had higher serum Gal-9 levels, compared to healthy control. This study proposed that plasma Gal-9 could be used as a novel biomarker for patients with influenza infection [56]. Taken together, these studies suggested that Gal-9 can alter the activities of the virus-infected cells and enhance the immune response, especially the Tim-3-expressing T cells to ameliorate influenza virus infection and pathogenesis.

## 4. Other Galectins

As we mentioned before, the galectin family is diverse, and they participate in the regulation of various physiological, biological, and immunological functions. Therefore, we analyzed and summarized the roles of galectins in influenza virus infection. Most studies reported that Gal-1, Gal-3, and Gal-9, which are categorized into different types of galectins, have regulatory effects to ameliorate influenza A virus infection using in vitro or in vivo models. Unfortunately, there is a lack of information regarding the other galectins classified into the same type with a similar structure. Whether these other galectins play certain roles in the regulation of influenza virus infection requires further investigation. 

## 5. Discussion

**Figure 4:**
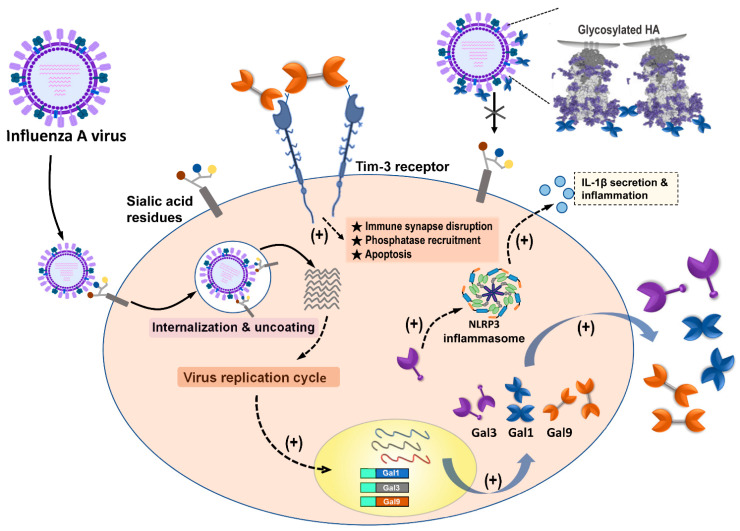
Summary of galectins against influenza A virus infection. Influenza A virus (IAV) infection triggered the induction and secretion of galectin-1 (Gal-1), galectin-3 (Gal-3), and galectin-9 (Gal-9). Secreted Gal-1 could bind to HA glycoprotein of IAVs and further blocking IAV interaction with sialic acid receptors expressed on the cells. IAV infection upregulated endogenous Gal-3, which could induce NLRP3 inflammasome activation, as well as IL-1β secretion, to result in inflammation occurrence. The secreted Gal-9 could interact with Tim-3 expressing cells. Gal-9/Tim-3 interaction triggered Tim-3 downstream signaling and induced apoptosis.

A systemic literature review was performed to analyze the current information regarding the regulatory and potential therapeutic roles of galectins in influenza virus infection. Our results indicate that among galectin members, only Gal-1, Gal-3, and Gal-9 were reported to have abilities to regulate influenza infection and replication via directly binding to glycosylated influenza HA or indirectly enhancing the immune response against IAV invasion (Figure 4) (Table 1). We also noted that out of the other type of influenza virus, only IAV had studies that reported its interaction, correlation, and modulation with galectins. Most studies provided lines of evidence denoting that expression or treatment with Gal-1 and Gal-9 ameliorated IAV infection via blocking viral binding and enhancing T-cell immune responses, respectively. The Gal-3 expression also participated in the amelioration of IAV infection; however, there were a few studies that offered data showing that Gal-3 had promotion capabilities during IAV and *S. pneumoniae* coinfection in vitro and in vivo models (Table 1). 

Galectins were initially discovered in 197, based on their galactoside-binding activity. Galectins reside in the cytosol or nucleus for the majority of their lifetime, and they reach their galactoside ligands only after nonclassical secretion that bypasses the Golgi apparatus [18]. Accordingly, many previous studies focused on studying the regulatory functions of CRD of galectins and their interaction with glycan or glycoconjugates expressed on the cell surface. However, instead of glycan–CRD interaction, the endogenous functions of galectins which exert their regulatory function via protein–protein interaction are gradually gaining attention. For example, Chen et al. reported that endogenous Gal-3 expression induced pulmonary inflammasome during H5N1 avian influenza infection via binding to NLRP3 and ASC and further enhancing ASC oligomerization and NLRP3 inflammasome activation [41]. This regulatory effect, induced by endogenous Gal-3 on the H5N1 virus, suggests a protein–protein interaction.

As we mentioned before, galectins are known to be involved in various biological and biophysical regulations, including defense against microorganisms [57]. Recently, galectins have been recognized as modulators and pattern recognition receptors (PRRs) in response to virus or bacterial attack [24,57,58]. Our study indicates that only a few galectins were reported to participate in the regulation of IAV infection, replication, and propagation. Despite there being 12 animal galectins identified in humans, only Gal-1, Gal-3, and Gal-9, characterized as the prototype, chimera, and tandem-repeat type of galectins, respectively, have been reported to have regulatory effects on IAVs. Each type of galectin family contains several members and each member in the same family are proposed to have similarities in sequence and structure as well as comparable regulatory capabilities [59]. Nevertheless, they might be some differences owing to the 20–40% identity of amino acids in the CRDs among the different galectins and 80–90% identity of the same galectin from different mammalian species [60]. We, therefore, suggest that there might be more galectins with regulatory capabilities toward IAV infection; hence, further investigation is required. Furthermore, while galectins have been wieldy studied in various fields, the discovery of galectins in virus research is still in its initial phase. 

To date, the conventional strategy for influenza treatment is the usage of antiviral drugs [11,61]. The two major antiviral drugs that are used for the clinical treatment of influenza include NA and M2 protein inhibitors, which inhibit NA activities and block M2 ion channels, consequently inhibiting virus budding and the vRNP release [11]. Unfortunately, the occurrence of influenza drug resistance causes a major problem for this influenza treatment strategy. Reports indicated that approximately 45% of all influenza A subtypes in circulation, globally, were resistant to the adamantanes by 2013 (>69% of H1 subtypes and 43% of H3 subtypes) [11]. Resistance to NA inhibitor shows a lesser increase in comparison with the adamantanes. Prior to 2007, oseltamivir resistance was 1–5% detected in clinical practice. During the 2008–2009 influenza season, several countries reported isolating high oseltamivir-resistant strains [62]. Fortunately, the 2009 pandemic (pdm09) influenza A H1N1 strain emerged globally, and most infected cases showed susceptibility to oseltamivir treatment [63]. Based on this information, we recommend that there is a demand for the development of a novel anti-influenza strategy to overcome influenza-drug resistance; thus, we propose that galectins may have the potential to be applied as an alternative anti-influenza treatment strategy.

Furthermore, our results indicated that Gal-1, Gal-3, and Gal-9 were upregulated after the IAV invasion. These galectins are ubiquitously expressed in various cells and are detected intracellularly, in the cytoplasm and the nucleus, as well as extracellularly, outside the environment. In addition, the influenza virion contains abundant glycosylated HA and NA on its envelope (about 350 to 400 HA trimers and 50 neuraminidase tetramers) [64]. The glycosylation of HA and NA were shown in various degrees among different subtypes or strains of the influenza virus. The HA molecules have glycosylation sites ranging between 5 and 11, whereas the NA molecule has three to five potential glycosylation sites [65,66]. Gal-1, Gal-3, and Gal-9 were all reported to bind to IAVs via the interaction of its CRD with viral glycans, but only Gal-1 and Gal-9 were reported to result in the inhibition of HA binding to sialic acid receptors on host cells. However, Gal-3 did not show the enhancing effect on influenza attachment and internalization via extracellularly adding route [41]. Nevertheless, the crosslink and multivalent activity also displayed by Gal-3 when interacting with glycan might suggest that comparable CRD–glycan interaction still has a chance to result in a different regulatory outcome. However, the detailed mechanism of this phenomenon remains unclear.

Here, we reported that Gal-3 regulated avian H5N1 virus infection via promotion of NLRP3 inflammasome activation to enhance pulmonary inflammation [41]. The avian H5N1 virus has been reported to be more virulent than the seasonal influenza virus and could induce severe pneumonia, immune dysregulation, and cytokine storm [67]. However, the detailed mechanism of avian H5N1-induced pathogenicity and higher mortality remains not fully understood. During influenza virus infection, NLR family members are known to play important roles to regulate antiviral responses, especially for NLRP3 inflammasome since it has been reported to mediate several virus infections via promoting antiviral immune responses [39]. Except for IAVs, several studies have demonstrated the essential role of endogenous Gal-3 in infection-induced inflammatory response against either virus or bacteria invasion via inducing neutrophil infiltration or proinflammatory cytokine production such as IL-1β, TNF-α, and IFN-γ [17]. Accordingly, we suggest that Gal-3 plays a role in the regulation of microbial infection by increasing inflammatory response.

Similarly, Gal-9 was also reported to be upregulated during IAV infection and exerted anti-influenza effects [51]. Our results denoted that Gal-9 and Tim-3 interaction resulted in the amelioration of IAV infection and replication. Previous reports indicated that Gal-9 binding to Tim-3 on T cells could limit the extent of immunopathological lesions in autoimmunity, as well as in some chronic infections [68]. From the immunological viewpoint, the host immune response to virus infection needs precise regulation to minimize tissue damage while still achieving defense. However, some bystander tissue damage usually occurs due to several host defenses, enhancing inflammatory reactions and destroying nearby cells. Gal-9 is reported to play an immunomodulatory role in various microbial infections via interaction with its receptor Tim3, suggesting its regulatory effect on virus mainly through inducing immunopathogenesis [50]. Although a report indicated that Gal-9 inhibitory effect on IAVs occurs by binding to the virus particle [51], currently, most studies supported that Gal-9/Tim-3 interaction and downstream signaling is the key factor to modulate several virus infections, including IAVs [50,55]. However, Gal-9/Tim-3 signaling was not only found in the macrophage since this interaction was also detected in NK, CD4^+^, CD8^+^, and FoxP3^+^ regulatory T cells. We, therefore, suggest that further investigations are required to understand Gal-9/Tim-3 interaction signaling and how Gal-9 affects IAV infection through regulating different immune cells.

Discovering novel or even alternative therapeutic strategies is necessary to overcome the limitations of the current use of conventional antiviral drugs and vaccines against influenza viruses. In this study, we suggest that galectins have anti-influenza capabilities and could be a potential candidate to develop an alternative influenza treatment and prophylactic control use.

## Figures and Tables

**Table 1:** Summary of the antiviral roles of galectins against influenza virus infection.

Galectin	Antiviral Effects	Target Virus	Ref.
	Gal-1 directly binds to the envelope glycoproteins of influenza virus and constrain the viral hemagglutination activity and infectivity.	A/WSN/1933(H1N1)	[28]
**Galectin-1**	Recombinant Gal-1 (rGal-1) treatment reduced mice fatality via mediating the expression of cytokines and chemokines.	2009 influenza A H1N1 subtype (H1N1pdm09)	[25]
	Gal-1 participated in regulation of cytopathic processes by H1N1pdm09 virus to induce an arrest of the cell cycle at the G0/G1 phase.	H1N1pdm09	[30]
	Gal-1 expression was correlated with the differential susceptibility to H7N9 influenza via extracellular matrix (ECM)-receptor interaction and mitogen-activated protein kinase (MAPK) signaling.	Human H7N9 isolates	[31]
	IAVs and *S. pneumoniae* coinfection induced the secretion of Gal-1 to the epithelial cell surface and further modulated the expression of SOCS1 and RIG1 and activate ERK, AKT, or JAK/STAT1 signaling pathways.	H1N1pdm09	[42]
**Galectin-3**	Aloe-emodin treatment ameliorated influenza H1N1 virus infection via up-regulation of Gal-3 expression to further trigger antiviral genes expression	A/Taiwan/CMUH01/2007(H1N1)	[38]
	Gal-3 enhances effects of H5N1 promoting host inflammatory response by up-regulating IL-1β via NLRP3.	A/Vietnam/1204/03	[41]
	IAVs and *S. pneumoniae* coinfection induced the secretion of Gal-3 to the epithelial cell surface and further modulated the expression of SOCS1 and RIG1, and activate ERK, AKT, or JAK/STAT1 signaling pathways.	H1N1pdm09	[42]
	Gal-3 preferred binding to desialylated multivalent glycoligands.	A/PuertoRico/08/1934 (H1N1)	[43]
**Galectin-9**	Gal-9 inhibited the infection of IAVs via Gal-9 binding to influenza virus particles to inhibit virus attachment.	A/Puerto Rico/8/34 (H1N1); Aichi/2/68 (H3N2); A/Hong Kong/483/97 (H5N1)	[51]
	Virus-specific CD8 T cells upregulate Tim-3 expression and Gal-9/Tim-3 interaction induce cell apoptosis after IAV infection from in vitro and ex vivo assays.	HK/×31 (H3N2) A/Puerto Rico/8/34 (H1N1))	[55]
	Influenza virus infection induces plasma Gal-9 expression, suggesting Gal-9 as a possible biomarker for influenza.	Seasonal influenza virus	[56]

## Data Availability

Not applicable.
